# Remote Sensing Methods for Flood Prediction: A Review

**DOI:** 10.3390/s22030960

**Published:** 2022-01-26

**Authors:** Hafiz Suliman Munawar, Ahmed W. A. Hammad, S. Travis Waller

**Affiliations:** 1 School of Built Environment, University of New South Wales, Kensington, Sydney, NSW 2052, Australia; a.hammad@unsw.edu.au; 2 School of Civil and Environmental Engineering, University of New South Wales, Kensington, Sydney, NSW 2052, Australia; s.waller@unsw.edu.au

**Keywords:** remote sensing, flood prediction, flood forecasting, flood hazard assessment, flood risk analysis, disaster management

## Abstract

Floods are a major cause of loss of lives, destruction of infrastructure, and massive damage to a country’s economy. Floods, being natural disasters, cannot be prevented completely; therefore, precautionary measures must be taken by the government, concerned organizations such as the United Nations Office for Disaster Risk Reduction and Office for the coordination of Human Affairs, and the community to control its disastrous effects. To minimize hazards and to provide an emergency response at the time of natural calamity, various measures must be taken by the disaster management authorities before the flood incident. This involves the use of the latest cutting-edge technologies which predict the occurrence of disaster as early as possible such that proper response strategies can be adopted before the disaster. Floods are uncertain depending on several climatic and environmental factors, and therefore are difficult to predict. Hence, improvement in the adoption of the latest technology to move towards automated disaster prediction and forecasting is a must. This study reviews the adoption of remote sensing methods for predicting floods and thus focuses on the pre-disaster phase of the disaster management process for the past 20 years. A classification framework is presented which classifies the remote sensing technologies being used for flood prediction into three types, which are: multispectral, radar, and light detection and ranging (LIDAR). Further categorization is performed based on the method used for data analysis. The technologies are examined based on their relevance to flood prediction, flood risk assessment, and hazard analysis. Some gaps and limitations present in each of the reviewed technologies have been identified. A flood prediction and extent mapping model are then proposed to overcome the current gaps. The compiled results demonstrate the state of each technology’s practice and usage in flood prediction.

## 1. Introduction

Recently, there has been an increase in natural as well as man-made disasters in the world. Hydrological extremities caused by human activities, increased urbanization, global warming, and weather change can be attributed to the dramatic rise in the global flood risks [1]. Among the natural disasters, flooding is the most devastating natural hazard. Floods are common in all parts of the world. However, their characteristics and intensity vary from region to region [2]. 

Apart from the destruction of infrastructure and agricultural lands, floods cause in-tense impacts on the people who may drown or who have severe injuries due to hypothermia, for instance [3]. Some additional fatalities may occur due to indirect effects of floods which include the destruction of the health infrastructure, the spread of infectious diseases, psychological distress, and starvation [4,5]. Being the most commonly occurring natural disaster, floods have caused approximately 53,000 fatalities on a global scale [4]. In Europe, the economic losses resulting from floods that occurred in the year 2005, 2007 and 2010 surpassed EUR 1 billion [5]. Floods that occurred in Europe in the year 2002 caused material damage worth EUR 20 billion [6]. The impact of floods on people and communities can be devastating. Similarly, in Australia, more than 900 fatalities occurred due to floods with an estimated cost of infrastructure damage to about AUD 5 billion [7]. Recent flooding in north-eastern China has caused catastrophic impacts in many cities within Shanxi province, including in its capital, Taiyuan, with 29 fatalities in the region. The event occurred over 14 days (1 to 14 October 2021), with the heaviest rainfall occurring over 5 days, from 2–7 October [8]. At 185 mm, the rainfall in Taiyuan over 12 h was the highest recorded rainfall in the area. This was seven times the pre-2010 overall average for the same month [9]. Around 1.76 million people were affected, and economic losses reached CNY 5.02 billion, or about USD 780 million [9].

Regions influenced by the disaster and the extent of damage can, in certain instances, go undetected due to the large geographical size of regions affected by rain and flood, which hamper immediate relief response activities [7]. Due to extent of the destruction, some regions become inaccessible, and the relief groups fail to provide their services. Currently, images of a disaster region are extracted with satellite and aerial imagery [10]. These images undergo various image processing techniques to make predictions regarding the possibility of flood occurrence in a particular region. The remote sensing technology extracts the characteristics of the flood region and gives information about the upcoming disaster and event challenges. With the help of obtained image and remote sensing data, flood risk maps can be produced. High-quality images are derived from remote sensing mechanisms such as synthetic aperture radar (SAR) technology that provides high-resolution images of the land and water reservoirs, even in bad weather conditions and low light. Using recent technologies such as artificial intelligence (AI) and image processing can assist in automatic flood risk mapping [10,11]. These methods can assist in the alarm systems and framework development that estimate the water level of a certain region and predict the upcoming floods. Coupling all these technologies with GPS increases the accuracy of results and provides the precise location of an upcoming disaster [11,12]. The use of such technologies for disaster prediction and management helps in lowering the destruction risk by issuing immediate warnings and formulating appropriate strategies for conducting emergency responses [11]. Through early disaster prediction, timely disaster response strategies can be adopted which include arranging for prompt evacuations and helping to build resilience in the long run. More awareness about flood-related disasters can be spread among the public. Communication gaps can be reduced, infrastructure failures can be avoided, and immediate contacts can be made with relief commissionaires [11]. Early planning and effective communication can help save mankind from hazardous situations. This can ultimately help in supporting economic and social development of the country [12]. 

Remote sensing technologies acquire data about objects and infrastructure on the surface of the Earth without being in direct contact by using various recording instruments [13]. Therefore, it is helpful in areas where no physical or close contact is possible [14,15,16]. Examples of such technologies include SAR, space-based imaging platforms, and satellites. This technique helps in faster data collection [17,18]. The data cannot be collected accurately with such efficiency using ground-based observations, whereas the remote-sensing technology gathers the same data by covering large spatial areas in the least time and provides a comprehensive view of the target objects [19]. It can capture images of distant objects despite bad weather conditions. The aerial photography and satellite images obtained using remote-sensing help in visualizing the topography and other terrain properties [17]. Such features help in locating natural disasters and evaluating their proportion. A wide variety of relief operations could be conducted by timely visualization of data obtained from the resources [20]. 

Several surveys have been conducted to review and evaluate the flood prediction systems [13], machine learning models for flood forecasting [13], disaster management using big data [14], flood mapping and damage assessment technologies [15]. A significant number of developed flood prediction systems use machine learning and image processing techniques for flood prediction. However, the use of various techniques and analyses associated with remote sensing for flood forecasting has not been assessed explicitly. Hence, a significant research gap was observed for assessing remote sensing-based technologies in the pre-disaster phase. Recent research in the field of remote sensing demonstrates its immense potential to make accurate predictions of an upcoming disaster and to play a vital role in flood risk analysis [18]. To overcome these research gaps, this paper presents a systematic literature review on the use of remote sensing techniques to manage floods in the pre-disaster phase by conducting accurate flood prediction. The classification framework presented in this paper categorizes the flood prediction technologies that use remote sensing based on data capturing and analytical approaches. The importance of each technique along with its current use, performance requirements, application and limitations have been highlighted to understand how it can be used to avoid the disastrous effects of floods in real-time by reliable flood prediction. This study also conducts a comprehensive comparison between these technologies based on various metrics [19,20,21]. The results of this study would help the concerned authorities in better understanding the use of technology for dealing with floods and choosing the most suitable model to manage floods in their target area. Precisely, this research is focused on answering the following research questions:

RQ-1. How can remote sensing technologies for flood detection be categorized?

RQ-2. How are remote sensing technologies being used for flood prediction?

RQ-3. What are the current gaps in remote sensing-based flood prediction technologies?

The rest of the paper is organized as follows: Section 2 describes the methods used to acquire materials to conduct this study, while Section 3 presents the results of the study by providing a comprehensive analysis of the remote sensing technique. Section 4 discusses the results of this research, identifies the research gaps, and presents a solution to overcome them. The paper closes with a conclusion in Section 5. Table 1 shows the list of acronyms used in the article.

## 2. Materials and Methods

The main aim of this paper is to conduct a comprehensive systematic review of the recent remote sensing technologies for flood prediction and to identify the existing research gaps when it comes to flood prediction. For this purpose, a detailed study of the literature based on this domain was carried out to identify recent developments in the timely prediction of floods. To achieve these objectives, the foremost step was to gather a set of the most relevant, recent, and authentic research articles published in top tier journals. We divide the search process of research articles into two main phases, which are: article retrieval and screening phases. In the following sections, we discuss each of these phases in detail.

**Figure 1:**
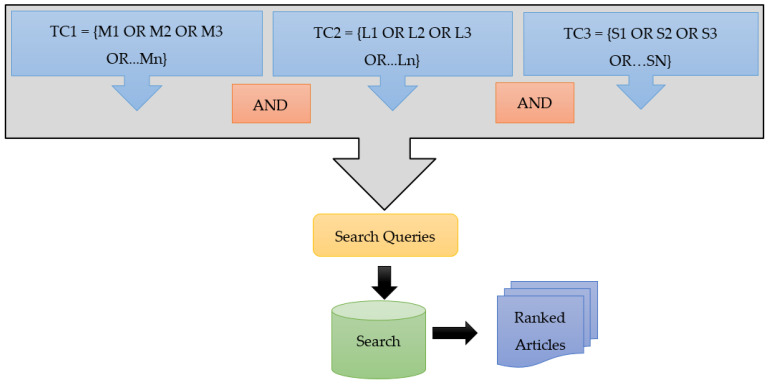
Query formulation for the retrieval of articles.

To retrieve the research articles for this study, the chosen search engines were Google Scholar, Web of Science, ERIC, IEEE Explore, Science Direct and Scopus. The next step was to formulate a set of queries to be used in each of these search engines to retrieve the articles. The major aim was to fully exhaust the search database and retrieve a maximum number of articles matching our domain of interest. We used three categories of terms representing the subdomains to extract a variety of research articles. The process of formulation of terms to be used as keywords in the search engines is demonstrated in Figure 1. In this figure, the notation TC denotes the term category to which the set of keywords or terms belongs. The AND operation indicates that the final set of search queries used in the search engine of each website must contain keywords from all three categories. After entering the search queries, a set of articles ranked based on their relevance were retrieved. The first category of phrases was formulated to retrieve articles that proposed flood prediction models using remote sensing technologies that utilized multispectral sensors. In Figure 1, “M” denotes the terms in this category. The phrases were formed by using keywords related to flood prediction which include “flood prediction”, “flood forecasting”, “flood risk analysis” and “flood hazard mapping” along with phrases such as “multispectral remote sensing” and “optical remote sensing”. The second category of terms was formulated to retrieve articles that proposed flood prediction methods using LIDAR remote sensing. For this purpose, we used flood prediction keywords along with the keyword “LIDAR”. In Figure 1, “L” represents the terms in this category. The third term category was aimed towards retrieving articles that used remote sensing technologies having radar sensors which include the Synthetic Aperture Radar (SAR). The search strings used to extract such articles used keywords specific to flood prediction along with the string “radar”. In Figure 1, “R” denotes the terms in this category. A total of 147 articles were retrieved after this search phase. 

**Figure 2:**
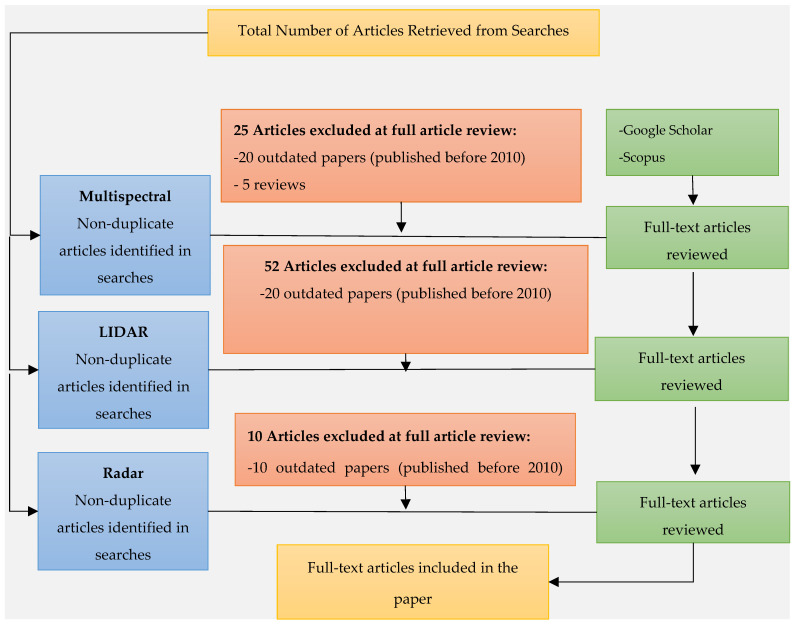
The Detailed Screening Process.

The number of articles retrieved from each category of search keywords is shown in Figure 2. The number of articles from each term category that passed the screening phase is shown in Figure 2. From the multispectral domain, initially, a total of 55 articles were retrieved because of the first phase. After analysing these papers based on screening criteria, 25 papers were excluded, resulting in the selection of 30 papers from this category. Similarly, 72 papers related to LIDAR were retrieved initially. This number was reduced to 20 after continuing through the second phase, as 52 articles were omitted. From the third term category, which is radar, initially, 30 papers were retrieved, out of which 20 passed the screening test. Hence, overall, 70 papers were finally collected as an output of the screening phase. 

**Figure 3:**
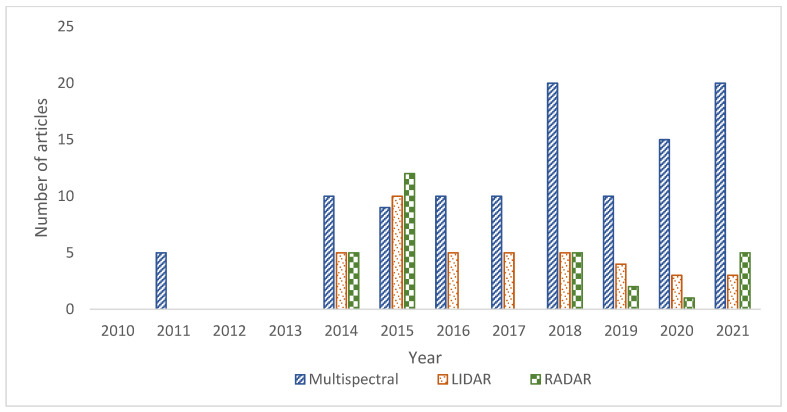
Year-wise distribution of articles from each category.

Figure 3 shows the year-wise distribution of articles retrieved from each category. This shows a significant increase in the use of multispectral remote sensing techniques for flood prediction as compared to radar or LIDAR based technologies in the past decade. Conversely, a comparatively smaller number of articles focused on the use of multispectral imaging technologies individually, for dealing with flood forecasting. An even smaller number of papers focused on radar-based remote sensing technologies. The search was extended to include reports, magazine articles, and web pages from authentic websites, thus increasing the scope and collecting a wide range of articles based on the subject matter. All the articles published before 1 January 2010, were discarded. This occurred to include the most recent technologies in the review. One exception to this rule was keeping some earlier papers that introduced basic concepts and definitions related to the technologies discussed in this study. This occurred to prevent any misrepresentation of information or modification of the key terms.

After the first phase based on article retrieval, the articles were passed through a screening phase to further narrow down the selection criteria. Four assessment criteria were defined to evaluate the articles:

1. No duplicates;
2. Time interval: 2010–2021;
3. Document type: research article, abstract, book chapter;
4. English language only

Thus, by filtering the articles based on these metrics, we were able to extract the most recent, applicable, and unique research articles written in the English language. From the 147 articles retrieved in the first phase, 70 articles passed all the four selection criteria. Hence, this review is based on these screened articles. 

## 3. Results

RQ-1. How can remote sensing technologies be categorized? 

**Figure 4:**
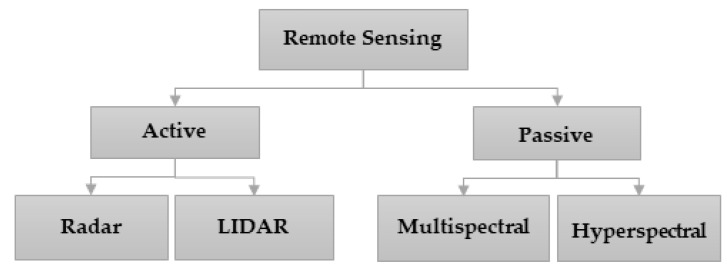
Classification of remote sensing methods.

As a result of the article retrieval and screening process, a total of 70 recent research articles were selected to be reviewed in this study. Remote sensing technologies can be categorized into two types which are active and passive. The active remote sensors use their light source to gain data from the target located on the surface of the Earth, while the passive remote sensing methods rely on natural light or the sun to gain this data. The technologies such as radar and LIDAR use their light sources; hence, they are categorized under the active class. The remote sensing methods that use satellites to capture imagery of the target on Earth can be classified as passive methods because the satellites rely on sunlight to capture these images. The satellite sensors may be able to capture multispectral or hyperspectral images. Hence, two further types of passive remote sensing can be defined, which are multispectral and hyperspectral. Figure 4 shows the classification framework of remote sensing technologies. Most remote sensing technologies belonged to the multispectral category and hence rely on satellites. Flood prediction models belonging to the multispectral, LIDAR and radar-based remote sensing domains are analysed in detail in the subsequent sections along with their advantages and potential drawbacks.

RQ-2. How are remote sensing technologies being used for flood prediction?

### 3.1. Multispectral

Multispectral remote sensing stores the emitted or reflected energy from objects present on the surface of the Earth through sensors that can recognize specific spectral bands [22]. The spectral bands form a thin portion of the electromagnetic spectrum, specified by the lowest and highest wavelength that is recognizable by the sensor. As a result, one raster image is saved for each of the spectral bands [22,23,24,25]. Examples of current satellites making use of such sensors include Sentinel-2, Landsat 7, Landsat 8, and MODIS. In this section, a review of these technologies for flood prediction is presented. Wieland and Martinis presented a framework to perform flood prediction on multispectral data gained from Landsat TM and Sentinel-2 images [26,27,28]. A convolutional neural network (CNN) was trained using these data to perform segmentation to determine water extent levels. Biases that may occur during downstream analysis are overcome by especially handling noise data such as clouds, shades, and frost. It outperforms the Random Forest classifier and a Normalized Water Index (NDWI) threshold function. Massari et al. [29] retrieved readings of soil moisture using the Advanced Scatterometer (ASCAT) to develop a rain-fall-runoff model that forecasts floods. The direct association between the satellite, soil moisture and rainfall are utilized in the model to make decisions regarding the future occurrence of floods. The study took place in the Mediterranean Sea, where readings from ten catchment locations in the ocean were recorded. These observations were acquired using the ASCAT satellite. These data are given as input to a rainfall-runoff calculation method called MISDc to obtain rainfall estimates. The rainfall data were used to predict the high-water flows in the Mediterranean Sea. Shahabi et al. [30] identified flood-prone areas using multispectral data acquired from the Sentinel-1 satellite of the Haraz watershed located in Iran. A machine learning-based ensemble method was used to perform flood susceptibility mapping. This model was composed of a combination of K-Nearest Neighbour (KNN), bagging, and a cubic classifier. Ten conditioning factors were gathered to train the model. Validation of the model showed that this ensemble method performs well and outperforms many other ensembles. The bagging approach significantly improved the accuracy of the KNN-cube ensemble for flood management and mapping problems. Noymanee [31] experimented with linear regression, ANN, boosted decision trees, Bayesian linear model, and decision forest to forecast floods in the district of Pattani in Thailand [31]. Bayesian Linear model demonstrated the best performance among all selected models, and was, therefore recommended for flood detection. A mathematical model was designed to model the upper and lower portions of the river stream. For example, to model the upstream part of a river, the following formula was used:

(1) $$W_{TX33*}=M\left(W_{TX347}\left(T\right),R_{TX347}\left(T\right)\right)$$

In the above equation, *M* represents the machine learning operation, *W* is the water level, the symbol * denotes predicted value, *TX*347 and *TX*33 are labels of the stations which are assigned to various river portions and R represent the rain value [31]. The flood mapping results in an input multispectral aerial image. The system classifies the flooded (Red) and non-flooded (Blue) regions and highlights them using different colours in the output such that the rescue workers can easily distinguish between them. Zhang et al. compared the flood prediction results for both spatial and temporal resolutions of existing sensors [32]. Landsat and MODIS images were collected for real-time prediction of floods. The models achieved a high level of accuracy which proved that for Landsat images both spatial and temporal models generate similar results for real-time prediction of floods. Cenci et al. evaluated the ability of Sentinel-1 to acquire soil moisture data for flood forecasting [33]. The soil moisture readings recorded by the satellite were used in a hydrological model called “Continuum” to predict flash floods. The study area was the Mediterranean Sea. The hydrological assimilation of different GEO SAR-like soil moisture products was evaluated using the SAR images. The results showed the effectiveness of Sentinel-1 derived soil moisture data to improve the flood predictions, especially for heavy flows. The Sentinel-1 data need the application of proper pre-processing methods before assimilating the data. Another finding was that apart from the need for high spatial resolution of the satellite, the temporal resolution of the satellite also plays an important role in the acquisition of correct data for the hydrological model. Ogilvie et al. [34] combined flood events data and satellite imagery to build a numerical model that monitors the water level in reservoirs. For this purpose, the rainfall run-off model and water level models were built for seven reservoirs. The data were collected between 1999 and 2014. An Ensemble Kalman Filter was applied to reduce the rainfall run-off errors and classification outliers. This method was able to reduce the root mean squared error by 54% when compared with flood forecast results provided by the previous hydrological model. Optical imagery was used for the measurement of water levels which helps in defining the scope of a flooded area [35]. The water level of a wide area can be measured in consecutive events. Analysing the change in water level can help in the easy prediction of flood events. This technology also takes the data of absolute water elevations. The data help to develop protocols for flood management and gives immense information for environmental science research. Remote sensing measures the accuracy of water up to decimeter level and shows real-time transmission [36]. Meng et al. [37] presented an approach to predict snowmelt floods in the Juntanghu watershed in China. A weather research and forecasting (WRF) model were used along with a snowmelt run-off model known as Tianshan Snowmelt Runoff Model (TSRM) which contains the snowmelt readings recorded during multiple years. Image data gathered from MODIS and DEM were used to predict floods using these hydrological models. The TSRM model driven by WRF was able to achieve 80% of condition ratios and determination coefficients of 0.85 and 0.82 for 2 years, respectively [37]. Boni et al. [38] combined data collected from Sentinel-1 and SAR to monitor floods in the Po River situated in Northern Italy. Image processing techniques such as thresholds, classification, and Region Growing Algorithm (RGA) were applied for the mapping of flood-prone areas [38]. The model achieved an overall user accuracy ranging from 60% to 80%. Li et al. used Sentinel-2 data along with data obtained from DEM having 90 m of spatial resolution. The noise data produced due to the presence of clouds, shadows, and frost were reduced using a Modified Normalized Difference Water Index (MNDWI), Revised Normalized Difference Water Index (RNDWI), Automated Water Extraction Index (AWEI), and Otsu threshold [39]. Google Earth Engine framework was used to calculate the water index and to extract water features. A root means a square error of 16.148 m was recorded using the proposed approach. Airborne SAR was studied for real-time flood area observation as well. Mason et al. [40] studied a method for selecting a subset automatically and in near real-time, which would allow the SAR water levels to be used in a forecasting model. Distributed water levels may be estimated indirectly along the flood extents in SAR images by intersecting the extents with the floodplain topography. It is necessary to select a subset of levels for assimilation because adjacent levels along the flood extent will be strongly correlated. Table 2 compares the performance outcomes and functionality of the multispectral remote sensing technologies used for flood prediction.

### 3.2. LIDAR

LIDAR stands for Light Detection and Ranging. It is an active remote sensing technology that uses laser pulses to measure the distance of an object present on Earth from the sensor [51,52,53]. The Lidar system records other data from the Earth’s surface and along with the returned light pulses, the data obtained are used to create a 3D model which represents the properties of the Earth’s shape and surface. A LIDAR system thus consists of a laser scanner and a GPS. This technology has been used in applications that monitor and examine the Earth’s surface. A more common application of LIDAR technology is to generate DEM to be used in GIS which facilitates the emergency response operations. Hence, it has an immense potential to monitor water levels in water bodies to predict any future occurrence of a flood. Recently, several research articles have proposed methods for flood risk assessment and prediction using LIDAR remote sensing. Webster et al. [54] employed LIDAR to acquire details related to the rise of sea level to produce food risk maps. LIDAR data are used to construct a Digital Surface Model (DSM) and DEM which show the ground and non-ground regions and highlight the elevated and normal sea levels in the study area. The results were validated using GPS technology which shows accuracy that exceeds 30 cm [54]. Lamichhane and Sharma [55] developed a flood warning system using a DEM derived from LIDAR. The acquired LIDAR data were also integrated with some field data related to flooding in the target area to determine the evacuation time required by the people. Flood risk maps were produced by an HEC-GeoRAS, a software that allows the processing of geospatial data in ArcGIS [55]. The flood risk maps were then combined with digital orthographic maps to construct a real-time online flood warning system for the public [56]. Fadi et al. [57] used three channels of geometrical data derived from LIDAR. The first channel consists of survey data, the second channel is based only on the data acquired by LIDAR and the third one consists of a combination of the riverbank locations derived from survey and cross-sections data acquired by LIDAR technology [57]. The study aimed to predict the return period of the storm in the target area. The data were processed in the HEC-RAS tool to make flood-related predictions. The results showed that geometries obtained from LIDAR predicted floods with higher widths as compared with the predictions made by survey-derived geometries. Makinano and Santillan [58] integrated data from several resources to construct an early flood warning system. These sources include LIDAR, an open-source flood model, meteorological data, real-time hydrologic data and geographic visualization tools [58]. The acquired data are used to construct a two-dimensional (2D) hydraulic model using the HEC-RAS tool that produces accurate flood risk maps and provide early flood warnings. Stoleriu et al. [59] used high-density LIDAR derived data to improve the accuracy of flood risk maps generated by DEM [59]. HEC-RAS software was used to construct the flood hazard maps. The system was used to predict the flood reoccurrence probabilities in the durations of 33, 100, and 1000 years. The system can measure water levels up to an accuracy of 0.5 m. Table 3 summarizes LIDAR technologies for flood prediction.

### 3.3. Radar

Radar (Radio Detection and Ranging) [60,61,62] was first used in the year 1940 by the navy department of America. As its name suggests, this remote sensing technology makes use of radio waves to find various characteristics of objects such as their direction, speed, location, and range. The organization of radar is composed of a transmitter that generates electromagnetic waves in the domain of radio or microwaves, an antenna for transmission, an antenna for receiving, a receiver, and a workstation that processes the object characteristics. The transmitter emits radio waves which are reflected by the object and then return to the receiver, where it is analysed by the processor to determine different object properties [61]. 

Once the detection through optical remotely sensed data fails, the synthetic aperture radar (SAR) comes into action. A high-resolution synthetic aperture radar (SAR) has been frequently used in the detection of areas affected by floods [63]. The technology provides real-time assessment of devastated and flooded areas. The prime quality of this technology is its penetration capacity to clouds, rain, and haze [64]. It does not matter whether it is a bad or drastic environment or too much sunlight, the technology provides effective expertise. The technology can easily distinguish between light and water. Radar uses microwaves; thus, flooding surfaces can be easily detected by its sensors. The flat surface of the water reflects the signals away from the sensor. This causes a decrease in the intensity of returned radiation as compared to the incident radiation causing a darker pixel in the image [65]. Thus, areas with water show dark pixels as compared to the pixels formed by the deflection through land areas.

Mitigation and management of floods require the analysis of the spatial extent and progressive pattern of remotely sensed images. The spatial extents of flooding are necessary to save lives and to avoid destruction. Combining this information with GIS and satellite data can help in estimating the damage caused by a flood [66]. Satellite transmissions involving microwaves revolutionized data extraction even in bad weather conditions and sunlight [38,39]. Data assimilation techniques facilitated the real-time integration of SAR-derived water levels and developed forecast models for disasters [67]. The integration of sensing data with data assimilation provided 3D reports of the flood used for the prediction of the flood as well as organizing the warning system for the flood. A problem faced by this technology is its inability to measure the long-term and real-time water level at fixed points. This is because of the orbital cyclic movement of the satellite. Thus, regardless of its high accuracy and real-time monitoring, it does not fit as the best technology for urban flood prediction. However, it works best for large water bodies including oceans and rivers [68]. Garcia-Pintado developed a flood prediction model that used SAR-derived water level observations in the Severn and Avon rivers situated in the United Kingdom (UK). The authors proved that by applying an Ensemble Transform Kalman Filter (ETKF) directly, some divergence in the filter was caused due to false correlations. To overcome this problem, a spatial filter localization method is proposed. Overall results showed that this model is feasible to work as an independent flood forecasting model that uses Earth Observations (EO) [69]. Table 4 summarizes radar technologies for flood pre-diction reviewed in this paper.

## 4. Discussion

**Figure 5:**
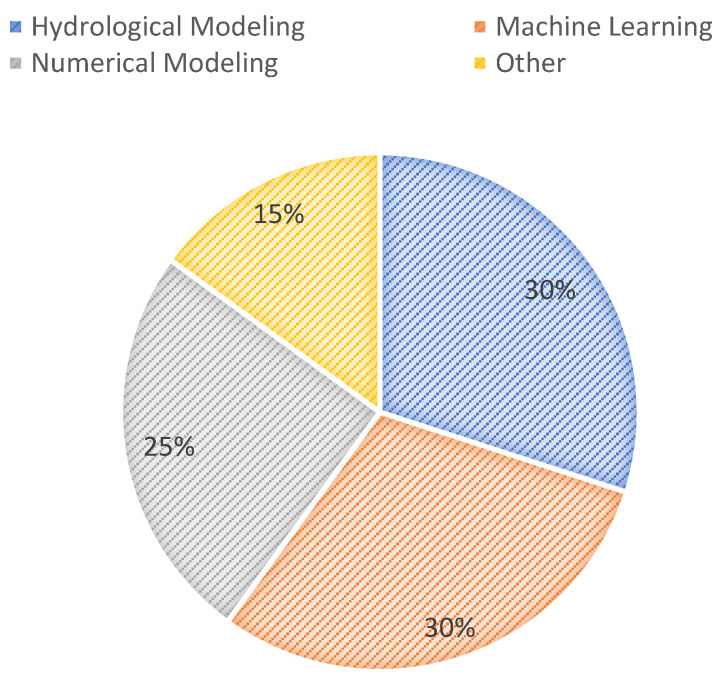
Methods adopted in studies.

Three categories of remote sensing technologies adopted for flood prediction are Multispectral, LIDAR, and Radar. After detailed content analysis and examination of the methods adopted in each study for flood forecasting, we further categorized these studies based on the processing method used by the authors. For example, the use of machine learning (ML) methods for flood prediction has been commonly found in the literature [44,45,46,47]. This includes the use of CNN, AI, KNN, Bayesian linear, SVM, etc. used for flood forecasting. Image segmentation has been applied to remotely sense images to determine water fluctuation levels to build an active real-time urban flood warning system [31]. Apart from that, many studies [45,49] have used hydrological models for flood forecasting using remotely sensed data. Numerical modelling techniques which include thresholds and filters have been commonly used in the literature [54,57]. The difference between machine learning-based methods and hydrological models is that machine learning methods are data-driven and mainly depend on the training data to produce accurate results, while hydrological models are knowledge-based which implies that the human experts already feed them the knowledge to make flood-related decisions. Numerical modelling is a commonly used technique in geology, which is used to solve complicated geological problems. Numerical modelling methods work by simulating geological states and scenarios. They use mathematical models to define the physical properties of scenarios related to geology using numbers, calculations, and equations. Other less commonly used domains included decision making [32], image processing [55], and electromagnetic modelling [41]. These domains are categorized into the “Other” class. Figure 5 shows the distribution of the reviewed flood prediction techniques to the identified domains. This pie graph shows that the use of machine learning and hydrological models has been equally and most frequently observed in the literature for flood forecasting.

RQ-3. What are the current gaps in remote sensing-based flood prediction technologies?

A significant issue in remote sensing technologies is the constraint regarding orbital cycles and spaces between trajectories of satellites, which makes the continuous monitoring of fixed objects a difficult task [31,71]. 

Floods, being natural disasters, are uncertain and unpredictable, which makes flood modelling a complex task having numerous uncertainties. Hence, hydrological and numerical models seldom provide imprecise results and fail to give reliable predictions regarding floods. In addition, these models are case specific, which means that they depend on the physical properties and climate of the specific study area [45,51,72]. Hence, they cannot be applied to a new area without modification. In addition, the high-resolution images captured by satellites are stored in satellite databases, making the processing slow and time consuming, with expected delays in the final output [53]. Image processing techniques, conversely, have some limitations regarding robustness and consistency, as the results are greatly affected by environmental conditions such as cloud cover, fog, rain, and pollution. Hence, most of the algorithms do not perform up to the mark in less-than-ideal conditions. To overcome these problems, researchers are rapidly adopting machine learning or artificial intelligence-based methods. The machine learning techniques are robust to the quality of input images, as these algorithms are trained using a wide variety of images having different illumination conditions, scale, quality, and colour, which enables the model to handle varying inputs. A limitation of machine learning models is that they must be trained using discriminative and relevant data, as the presence of noise and irrelevant information decreases the performance of the model [55]. In addition, these models require structured data that is the data must be labelled, which is an extensive and tedious task. Hence, there is an inherent need to efficiently extract features from the remotely sensed data and use it for training.

### Insights and Future Directions

Machine learning techniques manage the uncertainties related to natural disasters such as floods in an efficient way. The limitations of machine learning models can be overcome by providing reliable historic flood data and flood inventory maps to the machine learning model [31,73]. Machine learning models offer a cheap and time-efficient solution to predict floods and perform a flood risk assessment. They also inform the experts about the need for additional data such as data related to rainfall run-off and river flow. By providing this data, the model can generate more accurate prediction results.

**Figure 6:**
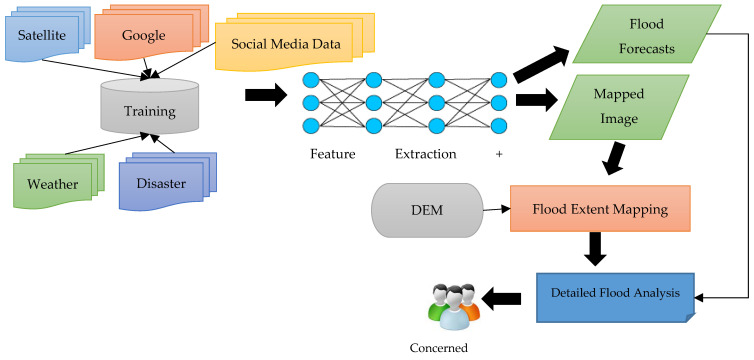
Schematic diagram of proposed system for flood detection and extent mapping.

The extensive labelling and feature extraction steps performed in machine learning methods can be reduced by using a deep learning approach. Deep learning models can use unstructured data and can automatically perform feature extraction. The research in this paper shows that researchers, to deal with disasters such as floods, have rarely adopted deep learning methods. Deep learning models have not been well experimented with or documented for flood risk analysis [42,74]. Hence, this domain needs to be further explored. This can be achieved by retrieving data from numerous sources, including disaster history, satellite imaging, and weather reports. The data gathered can be used to train the deep learning system. The deep learning model would be able to forecast the upcoming disaster events and be trained to perform real-time flood mapping. A proposed system for flood detection and extent mapping is demonstrated in Figure 6.

To overcome the issues faced by remote sensing technologies, the proposed system uses data from various sources along with satellite data such as past flood events, Google Earth Images, social media, and weather reports. The collected data would consist of both text and images, hence providing rich information about the nature, causes, locations, and effects of flood events. The data would be utilized to train a deep learning model such as CNN to predict future flood events and do real-time flood mapping in an input image. A team of experts can help in disaster management with the analysis reports produced by a deep learning system. In this way, proactive measures could be taken, and imminent devastation can be prevented. Natural disasters cannot be prevented; thus, one should take active steps to protect themselves from these situations. Using the deep learning system with drones such as UAVs, experts can gather real-time data from various areas and perform flood mapping at the same time. The drone technologies could track specific areas and deliver help in narrow territories. Deep learning, in today’s life, is ready to show its potential in disaster management. Classical machine learning models such as SVM, Naïve Bayesian, and Decision Trees require extensive steps of labelling the training data and selecting the relevant and discriminative feature for training the model. Deep learning models, conversely, have an inherent capability of efficiently handling unstructured data and automatically extracting features from it. Hence, the images in the training set do not have to be labelled and can be used directly for training. This saves time and reduces the complexity of the system. Some most common deep learning models are Convolutional Neural Networks (CNN), Recurrent Neural Networks (RNN), and Long Short-Term Memory (LSTM). CNN has been widely used for image classification and segmentation problems [43,75]. RNN models can efficiently handle temporal data and have been applied to video processing problems. LSTM model is an improved form of RNN, which can retain information for longer periods, unlike a standard RNN model. Hence, the future direction for using technological advancements for dealing with floods would be to investigate the use of deep learning for real-time flood mapping and prediction. To find the depth of floodwater in a region, DEM can be integrated into the system, such that rescue activities could be prioritized in the regions having deeper floodwater. The framework proposed in this study aims to bridge the gaps identified in various flood management technologies belonging to different domains. For real-time flood mapping in emergency scenarios, time is one of the most important factors to be considered, and the system should be efficient enough to map the flooded regions immediately, such that rescue operations can be initiated as soon as possible [76]. However, in most of the research studies, this measure was not evaluated in the performance results. The researchers should specify the time taken by their system to produce the results, as a slow and lagging system is not suitable to be implemented in an emergency. This would help in the better assessment of flood management technologies in the future. More research needs to be focused on using technologies to facilitate post-flood rescue and relief operations. This includes route finding, vehicle detection, and locating affected people such that the people stuck in flood-related crises could be identified and rescued by finding the available routes and transport facilities.

## 5. Conclusions

This article presents a systematic review of remote sensing technologies used for flood predictions. The review indicated that there is a rapid surge of studies implementing AI techniques coupled with remote sensing techniques for flood prediction. Based on a content analysis methodology, a review of 76 relevant papers on remote sensing technologies for flood prediction was presented. A classification framework for flood detection and mapping was proposed that aimed to answer the proposed questions: (1) How can remote sensing technologies for flood detection be categorized? (2) How are remote sensing technologies being used for flood prediction? (3) What are the current gaps in remote sensing-based flood prediction technologies?

To measure the depth of floodwater in a region, a high-resolution DEM can be used. Deep learning is a sub-domain of machine learning that uses neural networks that can undergo unsupervised learning from unstructured or unlabeled data. A framework has been proposed in this paper that uses a deep learning neural network to generate reports regarding the detection of flood from an input image and predict future flood events by learning from big data collected from various sources such as historic flood events, social media posts, Google and satellite images. A DEM module has been added to determine the extent of flooding in each area. This system is focused on disaster prediction and response. Detection of flooding from images can accelerate disaster relief services in the target region, thus assisting in the domain of disaster response. By finding the depth of floodwater in an area, rescue activities can be prioritized in the regions which have deeper flood water.

The outcomes of this research support the United Nations International Strategy for Disaster Reduction and Sendai Framework for Disaster Risk Reduction 2015–2030 [32]. As with the application of remote sensing technologies, the priority action of the Sendai framework can be met, which focuses on understanding the disaster risk, managing it, reducing disaster by building resilience and enhancing disaster preparedness through effective response and recovery. In addition, this study can assist the national disaster management authorities in the implementation of state-of-the-art technology for flood prediction, detection, and management. Other countries frequently hit by flood-like disasters can also benefit from this research. In the future, this study can be extended to include more techniques as well as identifying various domains, parameters and metrics for effective detection, prediction, and response to flood-related disasters and the assessment of various technologies. By defining a wide range of assessment measures, the techniques can be more thoroughly examined. We aim to implement the proposed flood management system proposed in real time to assess its limitations and practicability. 

## Figures and Tables

**Table 1:** List of acronyms used in the article.

Abbreviation	Full-Form
AI	Artificial Intelligence
ASCAT	Advanced Scatterometer
AWEI	Automated Water Extraction Index
CNN	Convolutional Neural Network
DEM	Digital Elevation Model
DSM	Digital Surface Model
ECV	Essential Climate Variables
ETKF	Ensemble Transform Kalman Filter
EO	Earth Observations
GIS	Geographic Information System
KNN	K-Nearest Neighbour
LIDAR	Light Detection and Ranging
LSTM	Long Short-Term Memory
MNDWI	Modified Normalized Difference Water Index
MISDc	Modello Idrologico Semi-Distribution in continuo
MODIS	Moderate Resolution Imaging Spectroradiometer
ML	Machine Learning
NDWI	Normalized Difference Water Index
RGA	Region Growing Algorithm
RNDWI	Revised Normalized Difference Water Index
RNN	Recurrent Neural Networks
SAR	Synthetic Aperture Radar
SVM	Support Vector Machine
TSRM	Tianshan Snowmelt Runoff Model
UK	United Kingdom
UAV	Unmanned Aerial Vehicle
WRF	Weather Research and Forecasting

**Table 2:** Multispectral remote sensing technologies for flood prediction.

Method	Domain	Features	Imaging Technology	Study Area	Results (% Accuracy)	Limitation	Ref
Convolutional Neural Network	Machine Learning	Water level Monitoring	Landsat TM, Sentinel-2	Bihar, India	Overall accuracy (OA) = 0.93 kappa (K) = 0.87	Need enhanced segmentation to support higher resolution imagery	[40]
Modello Idrologico Semi-Distribuito in continuo (MISDc) Model	Hydrological Modelling	Flood Forecasting	Metop A and B	Mediterranean Sea	Satellite-based data show improved results than ground-based data, with 100% efficiency	Results may be case specific	[41]
Bagging-Cubic-KNN	Machine Learning	Flood Susceptibility Mapping and detection	Sentinel-1	Haraz, Iran	The area under the ROC curve (AUC) = 0.80	-	[42]
Bayesian Linear	Machine Learning	Urban Pluvial Flood Forecasting	Sentinel-1	Pattani, Thailand	Improved results over a neural network and decision tree	Rainfall intensity found to be a weak predictor of floods	[43]
Support Vector Machines	Machine Learning	Urban Flood Mapping	MODIS & Landsat	New Orleans	Blending multisource images produce better results	Over or underestimation of flooded regions for urban areas	[44]
Continuum	Hydrological Modelling	Flash Flood Forecasting	Sentinel-1	Mediterranean catchment	Discharge Prediction results improved using Sentinel-1 data	Data recorded for a short period	[45]
Geodetically level gauge stations in water bodies	Hydrological Modelling	Modelling floodplain flow, processes, and fluxes in rivers	ICESat spaceborne Earth-orbiting laser altimeter	400km Amazon River	Accuracies of 10 ± 27 cm	Some uncertainties in results due to tidal influences	[46]
Generation of essential climate variables (ECV) for lakes	Numerical Modelling	Water surface-level variation calculation	Public web databases providing imagery of satellites instruments	-	Accuracy up to a decimeter level	-	[47]
WRF Model	Numerical Modelling	Flood Prediction	DEM, MODIS	Juntanghu Watershed, China	Determination Coefficients = 0.85, 0.82 for 2 years respectively	WRF Model	[48]
Classification, RGA, Thresholding	Image Processing	Mapping Flood Prone Areas	Sentinel-1, SAR, DEM	Po River, Italy	User Accuracy = 60–80%	-	[49]
MNDWI RNDWI AWEI Otsu Threshold Google Earth Engine	Hydrological Modelling	Open Surface River Extraction	Sentinel-2, DEM	Upper Yellow River, Tibetan Plateau	Root mean square error (RMSE) = 16.148 m	-	[50]

**Table 3:** LIDAR Technologies for Flood Prediction.

Method	Domain	Features	Imaging Technology	Study Area	Results (% Accuracy)	Limitation	Ref
**DEM**	3D Modelling	Flood risk mapping	LIDAR, GPS	Annapolis Royal, Nova Scotia, Canada	Accuracy > 30 cm	-	[23]
**DEM, HEC-GeoRAS**	3D Modelling, Hydraulic Modelling	Flood Warning System	LIDAR	Grand River near the City of Painesville, Ohio	RMSE = 0.37–0.98	Need accurate river streamflow data	[41]
**HEC-RAS**	Hydraulic Modelling	Flood return period prediction	LIDAR, Survey data	River Reach, Piedmont area of North Carolina.	LIDAR data predictions are 7% more accurate than survey data predictions	The method needs to be tested under varying climates	[51]
**HEC-RAS**	Hydraulic Modelling	Flood Forecasting & Early Warning	LIDAR	Philippines	Provide flood forecasts for the next 6–12 h	Method not backed by experiments and validation results	[52]
**HEC-RAS, DEM**	Hydraulic Modelling, 3D Modelling	Flood risk mapping	LIDAR	North-Eastern Romania	Accuracy = 0.5 m	Need high-resolution DEMs for accurate results	[53]

**Table 4:** Radar technologies for flood prediction.

Method	Domain	Features	Imaging Technology	Study Area	Results	Limitation	Ref
**Interferometric coherence for floodwater detection**	Electromagnetic Modelling	Real-time assessment of flooded regions	SAR	Urban Areas	Demonstrate successful flood mapping in the presence of wind	Concerns about the reliability of wind speed data	[70]
**Classification of global auxiliary data using fuzzy logic**	Machine Learning	Online Flood mapping	TerraSAR-X SAR, 175 images	Thailand and Germany	Thailand: 87.5%, Germany: 91.6%	Increased missed alarm rates due to noise	[49]
**Flash flood simulation using numerical methods**	Numerical Modelling	Flood Warning System	SAR	Dubuque, Iowa	Rainfall totals estimated comparable to the observed event	Need to develop a more generalized picture of the dynamics	[40]
**ETKF, Filter Localization**	Numerical Modelling	Flood Forecasting	SAR	Severn & Avon rivers, UK	Feasible to work as a standalone model	-	[57]
**PBD**	Hydrological Modelling	Flash Flood Forecasting	S-band NEXRAD (WSR-88D)	Austin City	RMSE = 0.89 m		[66]

## Data Availability

No new data were created or analysed in this study. Data sharing is not applicable to this article.
